# Andrographolide Protects against HG-Induced Inflammation, Apoptosis, Migration, and Impairment of Angiogenesis via PI3K/AKT-eNOS Signalling in HUVECs

**DOI:** 10.1155/2019/6168340

**Published:** 2019-10-07

**Authors:** Ming-Xia Duan, Heng Zhou, Qing-Qing Wu, Chen Liu, Yang Xiao, Wei Deng, Qi-Zhu Tang

**Affiliations:** ^1^Department of Cardiology, Renmin Hospital of Wuhan University, Wuhan, China; ^2^Cardiovascular Research Institute, Wuhan University, Wuhan, China; ^3^Hubei Key Laboratory of Cardiology, Wuhan, China

## Abstract

Andrographolide (Andr) is a major component isolated from the plant *Andrographis paniculata*. Inflammation, apoptosis, and impaired angiogenesis are implicated in the pathogenesis of high glucose (HG)-induced injury of vascular endotheliocytes. Our study is aimed at evaluating the effect of Andr on HG-induced HUVEC injury and the underlying mechanism. HUVECs were exposed to HG levels (33 mM) and treated with Andr (0, 12.5, 25, and 50 *μ*M). Western blot analysis, real-time PCR, immunofluorescence staining, the scratch test, and the tube formation assay were performed to assess the effects of Andr. We discovered that Andr inhibited the inflammatory response (IL-1*β*, IL-6, and TNF*α*), decreased the apoptosis ratio and cell migration, and promoted tube formation in response to HG stimulation. Andr ameliorated the levels of phosphorylated PI3K (p-PI3K), phosphorylated AKT (p-AKT), and phosphorylated eNOS (p-eNOS). The expression of vascular endothelial growth factor (VEGF) protein, a vital factor in angiogenesis, was improved by Andr treatment under HG stimulation. LY294002 is a blocker of PI3K, MK-2206 2HCI (MK-2206) is a highly selective AKT inhibitor, and L-NAME is a suppressor of eNOS, all of which significantly reduce Andr-mediated protective effects *in vitro*. Hence, Andr may be involved in regulating HG-induced injury by activating PI3K/AKT-eNOS signalling in HUVECs.

## 1. Introduction

Abundant evidence indicates that diabetes mellitus can cause a series of complicated pathophysiological changes including inflammation, apoptosis, autophagy, and a variety of vascular complications, which are the major risk factors for cardiovascular diseases [1, 2]. High glucose (HG), a primary independent risk factor for diabetic macrovascular disease, has been reported to play a key role in diabetes mellitus [3]. Many studies have suggested that HG not only triggers the proinflammatory response of adipose tissue in rats under a clamping environment but also causes apoptosis by increasing glucose oxidation and reactive oxygen species (ROS) levels [3, 4]. Angiogenesis is defined as the development of new blood vessels from existing capillaries or posterior capillaries through the migration and proliferation of endothelial cells. Evidence shows that HG suppresses angiogenesis due to lack of blood [5]. As the most potent angiogenic promoter, vascular endothelial growth factor (VEGF) increases vascular permeability and enhances endothelial cell proliferation to promote angiogenesis [6]. At present, the treatment of HG mainly focuses on a reasonable diet, proper exercise, and drug treatment. However, the prognosis of HG is still poor, and identifying novel therapeutic agents that inhibit inflammation and apoptosis and ameliorate endothelial dysfunction is important for HG induced injury [7].

Andrographolide (Andr) is the main effective component of the plant *Andrographis paniculata* [8]. This plant possesses various biological properties, including anti-inflammatory [9], antioxidant [10], antihypertrophic [11], and antiapoptotic [12] activities. Recent researches have reported the antihyperglycaemic effect of Andr. Liang et al. indicated that Andr treatment ameliorated diabetic cardiomyopathy via regulating NOX/Nrf2-mediated oxidative stress and NF-*κ*B-mediated inflammation [13]. Moreover, Andr inhibited the expression of fibronectin in diabetic nephropathy by suppressing the activation of activator protein-1 [14]. Andr inhibits HG-induced fibrosis and apoptosis and plays a protective role in murine renal mesangial cell lines [15]. The phosphoinositide 3-kinase (PI3K)/serine/threonine kinase (AKT) signalling pathway is involved in cell proliferation, migration, apoptosis, cell cycle, telomerase activity, and inflammation [16]. Extensive research has indicated that promoting PI3K/AKT signalling can suppress HG-induced inflammation [17], apoptosis [18], and impairment of angiogenesis [19]. Andr can regulate different cell types, such as rat primary hepatocytes [20] and human umbilical vein endothelial cells (HUVECs) [21], due to its ability to activate the PI3K/AKT signalling pathway. Based on these findings, we hypothesized that Andr may play a protective role in HG-induced injury by regulating PI3K/AKT signalling in HUVECs. The purpose of our study was to investigate the effects of Andr on HG-induced cell injury and the underlying mechanisms.

## 2. Materials and Methods

### 2.1. Chemicals and Reagents

Andrographolide (>98% purity) was obtained from Shanghai Winberb Medical Co. (Shanghai, China) and dissolved in a vehicle (DMSO, D2650). DMSO was purchased from Sigma-Aldrich (St. Louis, MO, United States). LY294002 (HY-10108), MK-2206 (HY-10358), L-NAME (HY-18729A), and cell counting kit (CCK)-8 were obtained from MedChemExpress (United States). The LDH and MDA assay kits were purchased from Nanjing Jiancheng Bioengineering Institute (Nanjing, China).

### 2.2. HUVEC Culture

The HUVEC-12 cell line (YRGene, NC006) was incubated with DMEM (1x) supplemented with 10% FBS, and the cells were cultured with 5% CO_2_ at 37°C for 48 hours. HUVECs between the third and fifth passages were used in our study [22, 23]. To assess the effect of Andr on HG-induced injury of HUVECs, the cells were seeded in 6-well, 24-well, and 96-well plates for 24 hours. After 4-6 hours of starvation in a serum-free medium, the cells were treated with Andr (12.5, 25, and 50 *μ*M) for 24/36 hours in HG (33 mM) or mannitol (MG; 27.8 mM mannitol+5.5 mM glucose). Cells were also treated with LY294002 (10 *μ*M) to block PI3K and MK-2206 (100 nM) to inhibit AKT and L-NAME (100 *μ*M) to suppress eNOS. After 24 hours, the cells in 6-well plates were collected for Western blots and RNA detection, the cells in 24-well plates were collected for TUNEL staining, and the cells in 96-well plates were collected for cell counting kit-8 (CCK-8) assay.

### 2.3. CCK-8 Assay

Cell viability was detected by the CCK-8 assay based on the manufacturer's instructions (Dojindo Molecular Technologies, Rockville, USA). HUVECs were seeded into 96-well plates at a density of 4500 cells/well. Cells were treated with Andr (12.5, 25, and 50 *μ*M) for 6-36 hours with/without HG, LY294002 (10 *μ*M), MK-2206 (100 nM), and L-NAME (100 *μ*M). Then, 10 *μ*M of CCK-8 was added to each well for 3 hours of incubation. The absorbance at 450 nm (OD450) was measured using an enzyme labelling apparatus, as described previously [11, 24, 25].

### 2.4. Detection of Lactate Dehydrogenase (LDH) and Malondialdehyde (MDA) Levels

LDH and MDA assay kits (Nanjing Jiancheng Bioengineering Institute, Nanjing, China) were used in accordance with the manufacturer's instructions to detect LDH and MDA activity. HUVECs were incubated in 24-well plates and treated with Andr (50 *μ*M), LY294002 (10 *μ*M), MK-2206 (100 nM), and L-NAME (100 *μ*M) for 24 hours. Then, the cells were centrifuged in tubes to obtain the supernatant. The ratio of cell number to extract was 500-1000 : 1, ultrasound was used to lyse the cells, and centrifugation was performed to obtain the supernatant. The absorbance was measured at 490 nm.

### 2.5. Quantitative Real-Time Polymerase Chain Reaction (RT-PCR)

HUVECs were seeded in 6-well plates for 24 hours. After 4-6 hours of starvation in a serum-free medium, the cells were treated with Andr (12.5, 25, and 50 *μ*M) for 24 hours in HG DMEM (1x). Total RNA was extracted from HUVECs using TRIzol (Invitrogen, USA), and the total RNA was reverse transcribed to cDNA using a reverse transcription kit (Roche, Germany) as reported previously [26]. The Roche LightCycler480 real-time PCR instrument (Roche, Germany) was used for the amplification, and the PCR results were quantitatively analysed. The mRNA expression levels of IL-1*β*, IL-6, and TNF*α* were normalized to GAPDH gene expression. The primers used in this study are shown in Table 1.

### 2.6. Western Blot Analysis

HUVECs were homogenized in RIPA lysis (Wuhan Google Biological Technology Co., Ltd, China) buffer to obtain total proteins. According to the previous studies [11, 17], the protein concentrations were measured by a BCA Protein Assay Kit (Thermo Fisher Scientific, USA) and denatured at 72°C for 10 minutes. Approximately 50 *μ*g of protein was separated by 10% SDS-PAGE and then transferred to PVDF membranes (Millipore, USA). After incubation with the specified primary antibodies for 12 hours at 4°C and secondary antibodies for 60 minutes at homeothermy, the membranes were washed three times with TBST. Then, the blots were visualized using a two-colour infrared imaging system (Odyssey, USA). Expression levels of proteins include VEGF (1 : 1000, ab46154, Abcam), p-PI3K (1 : 1000, 4228S, CST), T-PI3K (1 : 1000, 4245, CST), p-AKT (1 : 1000, 4060, CST), T-AKT (1 : 1000, 4691, CST), p-eNOS (1 : 1000, ab195944, Abcam), T-eNOS (1 : 1000, sc-654, SANTA), Bcl-2 (1 : 1000, 2870, CST), Bax (1 : 1000, 2722, CST), C-caspase3 (1 : 1000, 9661, CST), and GAPDH (1 : 1000, 2118, CST). These proteins were normalized to GAPDH expression.

### 2.7. TUNEL Staining

The cultured cells were prepared and stained according to the manufacturer's instructions, and the apoptotic cells were measured by the Apoptosis Fluorescein Detection Kit (Millipore, USA). Briefly, the cells were washed 3 times with PBS and fixed in 4% paraformaldehyde for 5 minutes. Cells were treated with proteinase K (20 *μ*g/ml) for 20 minutes and incubated with terminal deoxyribonucleotidyl transferase and deoxyuridine triphosphate for 60 minutes. Nuclei were stained with DAPI (Invitrogen, USA). The results were observed using a fluorescence microscope (OLYMPUS, Japan) and calculated using Image-Pro Plus 6.0 software (Maryland, USA).

### 2.8. Detection of Cell Migration and Tube Formation

The cells were seeded in 6-well plates and grew to cover the bottom of the plate. After 4-6 hours of starvation in serum-free medium, HUVECs were scraped with a 100 *μ*l sterile micropipette tip [22]. Next, the cells were treated with Andr (50 *μ*M), LY294002 (10 *μ*M), MK-2206 (100 nM), and L-NAME (100 *μ*M). Then, at 0, 12, and 24 hours, migratory cells were observed by an inverted microscope (OLYMPUS, Japan) and analysed by using by Image-Pro Plus 6.0 software (Maryland, USA).

According to the manufacturer's instructions, frozen BD Matrigel™ (Growth Factor Reduced, #356231) was melted into liquid at -4°C, and 10 *μ*l of Matrigel was added to the bottom of each ibidi vasculogenic glass slide. After the Matrigel solidified, the cell suspension (10^4^ cells/well) was added, and the cells were treated with HG for 24 hours. An inverted microscope was used to evaluate tube formation. Image-Pro Plus 6.0 software (Maryland, USA) was used to determine the number of tubes.

### 2.9. Statistical Analysis

All results are presented as the mean ± SEM and were analysed using SPSS 17.0 software. Comparisons between two groups were performed using an unpaired Student's *t*-test. One-way ANOVA was used for comparisons among three or more groups. A value of *P* < 0.05 was regarded as statistically significant.

## 3. Results

### 3.1. Andr Attenuates HG-Induced Inflammation and Apoptosis in HUVECs

**Figure 1:**
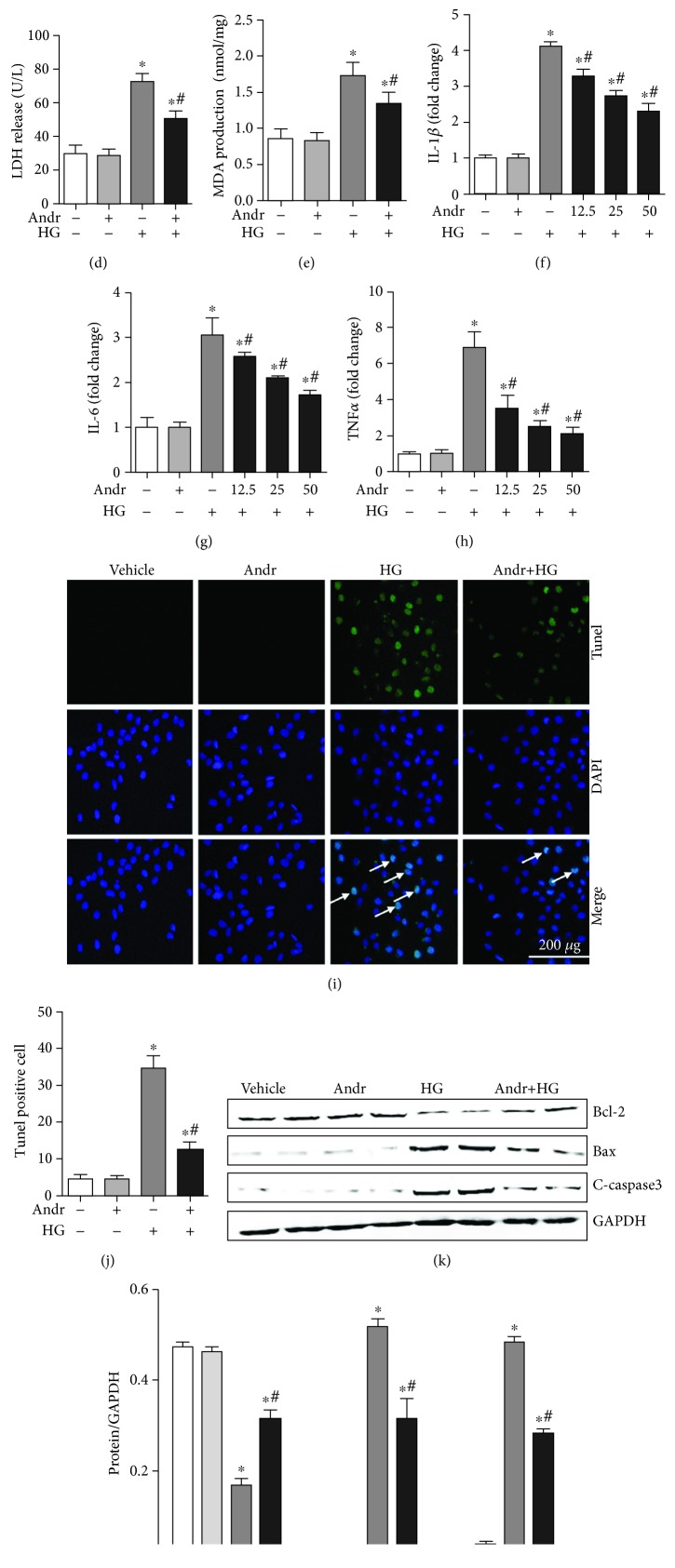
Andr attenuates HG-induced inflammation and apoptosis in HUVECs. HUVECs were exposed to HG (33 mM) and treated with different concentrations of Andr (0, 12.5, 25, and 50 *μ*M) for 24/36 hours. (a, b) The cell counting kit-8 assay was used to assess cell viability in each group (*n* = 6). (c) The effect of Andr was assessed at different time points in each group (*n* = 6). (d, e) LDH leakage (d) and MDA concentration (e) in each group (*n* = 6). (f–h) The mRNA levels of IL-1*β*, IL-6, and TNF*α* in HUVECs in each group (*n* = 6). (i, j) TUNEL staining and quantitative analysis of apoptotic cells in each group (*n* = 6). (k, l) The expression of Bcl-2, Bax, and C-caspase3 protein and quantitative analysis in each group (*n* = 6). ^∗^*P* < 0.05 vs. the control group. ^#^*P* < 0.05 vs. the HG group.

HUVECs were stimulated with HG and treated with different concentrations of Andr (0, 12.5, 25, and 50 *μ*M) for 24 hours. Four concentrations of Andr had no effect on cell viability (Figure 1(a)). Andr treatment improved the HG-induced decline of cell viability in a dose-dependent manner (Figure 1(b)). We also detected the beneficial effect of Andr at different time points (4, 6, 12, 24, and 36 hours), showing that the cell viability began to increase after 6 hours, reaching the highest level at 24 hours and remaining unchanged at 36 hours; thus, our experiment was conducted at 6-24 hours (Figure 1(c)). To evaluate the protective role of Andr under HG conditions, LDH leakage, MDA concentration, mRNA expression of inflammation biomarkers (IL-1*β*, IL-6, and TNF*α*), and apoptosis were detected in each group. The release of LDH and MDA, two indicators of HG-induced cell injury, was enhanced with HG stimulation and improved after 50 *μ*M Andr treatment (Figures 1(d) and 1(e)). Andr inhibited HG-induced inflammatory cytokine transcription in a dose-dependent manner (Figures 1(f)–1(h)). The HG-induced increase in the apoptosis ratio in HUVECs was attenuated by Andr treatment (Figures 1(i) and 1(j)). In addition, Andr decreased the expression of the proapoptosis proteins Bax and C-caspase3 and increased the expression of the antiapoptosis protein Bcl-2 after HG stimulation (Figures 1(k) and 1(l)). These data indicated that Andr plays a protective effect in response to HG injury, including inflammation and apoptosis.

### 3.2. Andr Attenuates HG-Induced Cell Migration and Impairment of Angiogenesis in HUVECs

**Figure 2:**
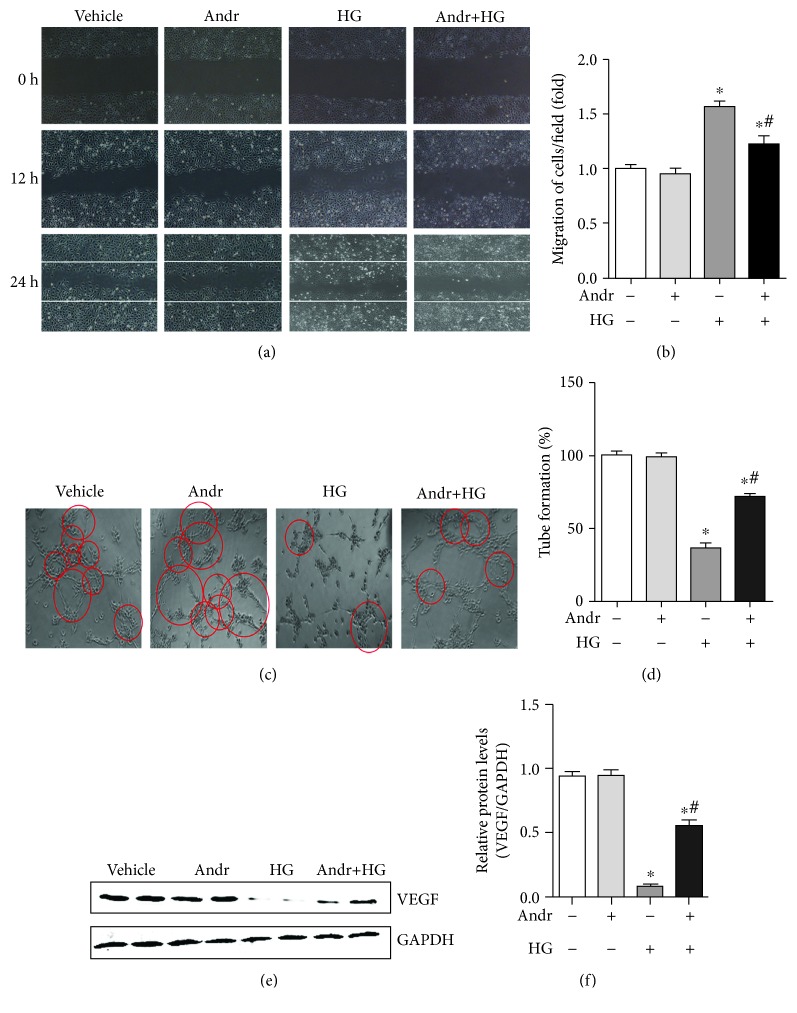
Andr attenuates HG-induced cell migration and impairment of angiogenesis in HUVECs. (a, b) In the scratch assay, HUVECs were treated with Andr (50 *μ*M) and/or HG for 24 hours and photographed at 0, 12, and 24 hours (*n* = 6). (c, d) In the tube formation assay, HUVECs were treated with Andr (50 *μ*M) and/or HG. After 24 hours, images were acquired by inverted microscopy, and the results were analysed in each group (*n* = 6). (e, f) The expression of VEGF protein and the quantitative analysis in each group (*n* = 6). ^∗^*P* < 0.05 vs. the control group. ^#^*P* < 0.05 vs. the HG group.

HG often leads to impaired angiogenesis [5] and promotes migration [21]. To evaluate the effect of Andr on migration and angiogenesis, we detected cell migration and tube formation in HUVECs exposed to HG with/without Andr treatment. Twenty-four hours after HG stimulation, HUVECs showed a large increase in cell migration (Figures 2(a) and 2(b)) and a decrease in tube formation (Figures 2(c) and 2(d)). Additionally, after 24 hours of treatment with Andr (50 *μ*M), the number of migrated cells was inhibited, and tube formation was substantially improved compared to that in the HG group. We also found that the expression of VEGF, a critical factor of angiogenesis, was significantly reduced with HG exposure. However, VEGF protein expression was noticeably ameliorated after Andr treatment (Figures 2(e) and 2(f)). These data showed that Andr could restrain HG-stimulated HUVEC migration and improve tube formation.

### 3.3. Andr Regulates PI3K/AKT-eNOS Signalling *In Vitro*

**Figure 3:**
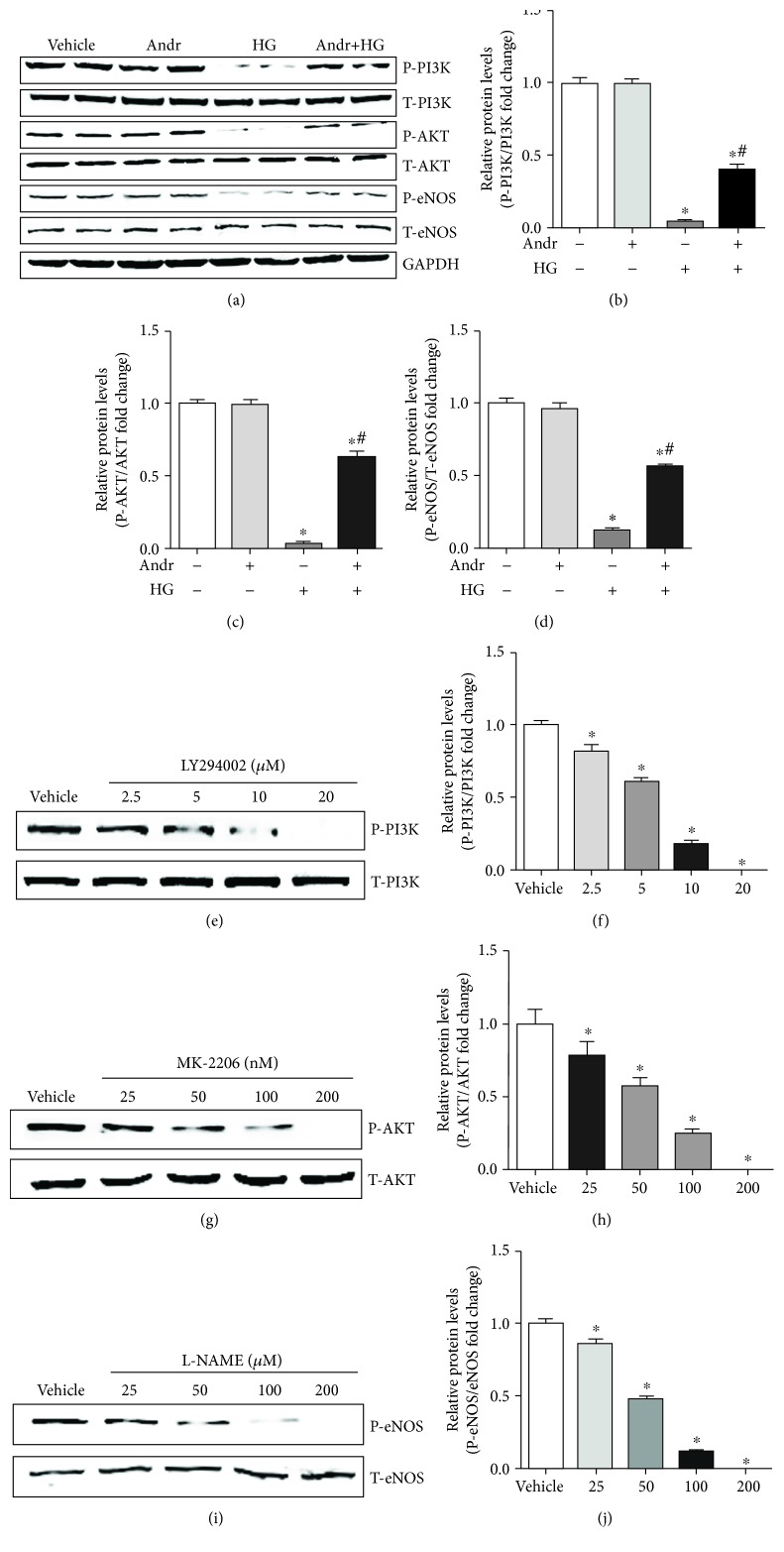
Andr regulates PI3K/AKT-eNOS signalling in vitro. (a) The expression of P-PI3K, T-PI3K, P-AKT, T-AKT, P-eNOS, and T-eNOS protein in each group (*n* = 6). (b–d) Quantification analysis of p-PI3K/PI3K protein, p-AKT/AKT protein, and p-eNOS/eNOS protein. (e, f) Effects of LY294002 at different concentrations on P-P13K and quantitative analysis of p-PI3K/PI3K protein (*n* = 6). (g, h) Effects of MK-2206 at different concentrations on P-AKT and quantitative analysis of p-AKT/AKT protein (*n* = 6). (i, j) Effects of L-NAME at different concentrations on P-eNOS and quantitative analysis of p-eNOS/eNOS protein (*n* = 6). ^∗^*P* < 0.05 vs. the control group. ^#^*P* < 0.05 vs. the HG group.

To assess the underlying mechanism of Andr in HUVECs under HG conditions, PI3K/AKT-eNOS signalling was detected. As shown in Figures 3(a)–3(d), the expression of p-PI3K, p-AKT, and p-eNOS proteins was decreased after HG stimulation for 24 hours, but the expression of T-PI3K, T-AKT, and T-eNOS proteins showed no significant changes. Andr increased the levels of p-PI3K, p-AKT, and p-eNOS protein and had no remarkable effects on T-PI3K, T-AKT, and T-eNOS. These data demonstrated that the protective role of Andr under HG conditions may be ascribed to the regulation of PI3K/AKT-eNOS signalling. As shown in Figures 3(e) and 3(f), LY294002 administration decreased P-PI3K levels in a dose-dependent manner (2.5, 5, 10, and 20 *μ*M). Figures 3(g) and 3(h) demonstrate that MK-2206 treatment decreased P-AKT levels in a dose-dependent manner (25, 50, 100, and 200 nM), and Figures 3(i) and 3(j) demonstrate that L-NAME treatment decreased P-eNOS levels in a dose-dependent manner (25, 50, 100, and 200 *μ*M). In the present study, we used LY294002 (10 *μ*M), MK-2206 (100 nM), and L-NAME (100 *μ*M) at the optimal concentrations.

### 3.4. Andr Attenuated HG-Induced Inflammation, Apoptosis, Cell Migration, and Impairment of Angiogenesis by PI3K-AKT-eNOS Signalling *In Vitro*

**Figure 4:**
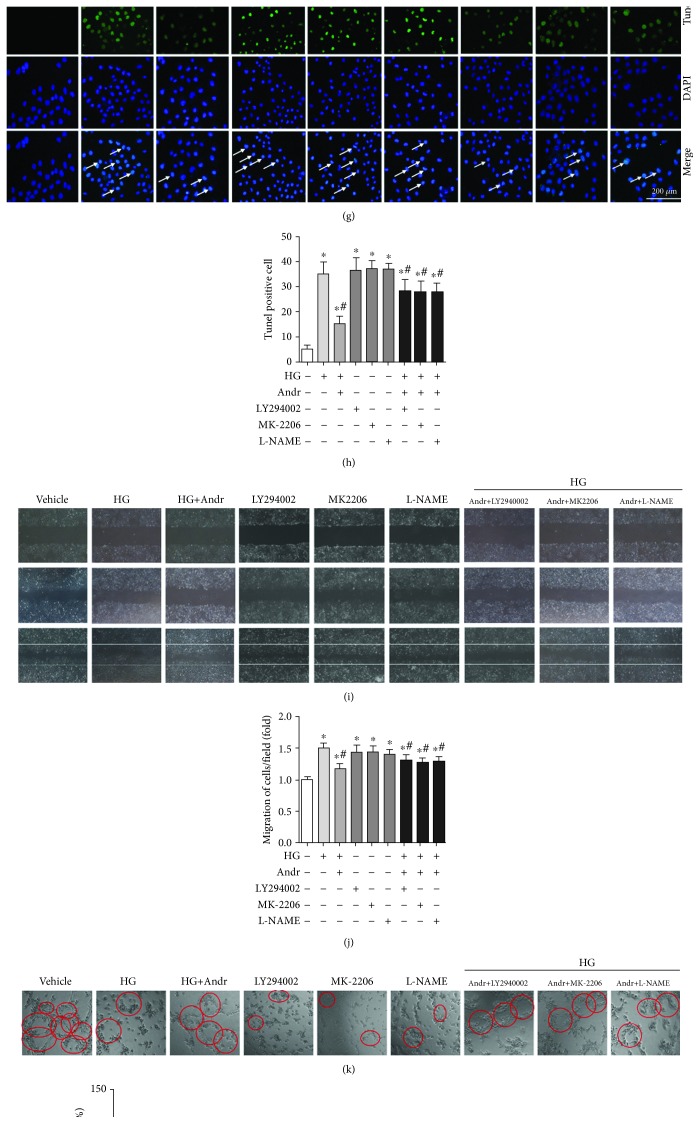
Andr attenuates HG-induced inflammation, apoptosis, cell migration, and impairment of angiogenesis by PI3K/AKT-eNOS signalling in vitro. HUVECs were stimulated with HG and treated with Andr (50 *μ*M), LY294002 (10 *μ*M), MK-2206 (100 nM), and L-NAME (100 *μ*M) for 24 hours. (a–c) Cell viability, MDA concentration, and LDH leakage in each group (*n* = 6). (d–f) The mRNA levels of IL-1*β*, IL-6, and TNF*α* in HUVECs in each group (*n* = 6). (g, h) TUNEL staining and quantitative analysis of apoptotic cells in each group (*n* = 6). (i, j) Scratch assay and the number of migrated cells in each group (*n* = 6). (k, l) Tube formation and quantitative analysis in each group (*n* = 6). (m, n) The expression of VEGF protein and quantitative analysis in each group (*n* = 6). ^∗^*P* < 0.05 vs. the control group. ^#^*P* < 0.05 vs. the HG group.

To further evaluate whether the effect of Andr relies on PI3K/AKT-eNOS signalling, we treated HUVECs with a PI3K inhibitor (LY294002), an AKT inhibitor (MK-2206), and an eNOS inhibitor (L-NAME) for 24 hours. As shown in Figures 4(b) and 4(c), HG-induced increases in LDH release and MDA production were markedly decreased in Andr-treated HUVECs, and the protective effect of Andr was significantly reduced after treatment with LY294002, MK-2206, and L-NAME. Meanwhile, in the LY294002, MK-2206, and L-NAME groups, the levels of LDH and MDA were markedly increased. Furthermore, LY294002, MK-2206, and L-NAME treatments significantly reduced the ability of Andr to enhance cell viability following HG stimulation (Figure 4(a)). Moreover, HG-induced inflammation and apoptosis, as evidenced by the increased mRNA levels of IL-1*β*, IL-6, and TNF*α*, as well as the number of apoptotic cells, were ameliorated by Andr but not by treatment with LY294002, MK-2206, and L-NAME (Figures 4(d)–4(h)). These data suggest that Andr exerts anti-inflammatory and antiapoptotic effects and may rely on the regulation of PI3K/AKT-eNOS signalling. We also performed cell migration and tube formation assays. LY294002, MK-2206, and L-NAME were used, and we observed that the number of tubes formed (Figures 4(k) and 4(l)) and the expression of VEGF (Figures 4(m) and 4(n)) were increased slightly; in addition, the number of migrated cells was decreased slightly by Andr treatment compared with that in the HG group (Figures 4(i) and 4(j)). These data indicate that the ability of Andr to protect against impaired angiogenesis and inhibit cell migration may depend on PI3K/AKT-eNOS signalling.

## 4. Discussion

Currently, chronic hyperglycaemia can not only directly cause injury of endothelial cells but also induce apoptosis by increasing the level of ROS and advanced glycation end products [27, 28]. An increasing number of studies have indicated that inflammation, apoptosis, and impaired angiogenesis contribute to the development of HG-induced injury [2, 5]. Andr is the main ingredient extracted from the traditional herbal medicine *Andrographis paniculata.* Previous studies demonstrated that Andr pharmacologically attenuated inflammation [9], hyperglycaemia [29], cardiac hypertrophy [11], and apoptosis [12]. However, the role of Andr in HG-induced HUVEC injury remains unclarified. In the present study, we found that Andr attenuates HG-induced inflammation, apoptosis, impairment of angiogenesis, and inhibition of migration in HUVECs. The PI3K inhibitor (LY294002), AKT inhibitor (MK-2206), and eNOS inhibitor (L-NAME) were used to block PI3K/AKT-eNOS signalling, and proinflammatory cytokine (IL-1*β*, IL-6, and TNF*α*) transcription and the number of apoptotic cells were inhibited by the three suppressors cocultured with those in the HG group. Nevertheless, HUVEC migration was inhibited, while HG-induced tube formation was restored. Thus, our study suggested that Andr alleviates HG-induced HUVEC injury by activating PI3K/AKT-eNOS signalling.

Long-term hyperglycaemia in diabetes gives rise to inflammation and cell death [30]. Research has shown that inflammatory cytokines are markedly increased in animal models of diabetic cardiomyopathy [30, 31]. A previous study revealed that Andr alleviated HG-mediated inflammation through AKT/NF-*κ*B signalling in diabetic nephropathy [10]. Moreover, the ability of Andr to attenuate cell apoptosis in high-fat diet-fed mice is due to enhanced IGF1R/PI3K/AKT signalling [12]. In our study, Andr decreased the mRNA levels of IL-1*β*, IL-6, and TNF*α*, inhibited the number of positive apoptotic cells, and downregulated the expression of apoptosis-related proteins, which is in accordance with the previous studies [9, 10, 21]. After treatment with LY294002, MK-2206, and L-NAME, inflammation and apoptosis were slightly improved by Andr compared with those in the HG group. Additionally, cell viability began to increase after 6 hours of Andr treatment with HG stimulation, reaching the highest value at 24 hours but remaining unchanged at 36 hours, which may be partly due to the use of 10% serum in our study. A previous study indicated that the medium containing low concentrates (2, 5, or 10%) of serum did not affect cell viability under normal glucose or HG conditions after 36 hours, which is consistent with our study [25]. On the basis of these findings, Andr restores HG-induced HUVEC injury due to its anti-inflammatory and antiapoptotic activities.

Chronic hyperglycaemia causes vascular endothelial cell dysfunction, which is regarded as one of the initial markers in diabetes [32]. LDH and MDA, two markers of HUVEC injury under HG conditions, were decreased after Ad-netrin-1 treatment [23]. Here, we found that Andr can reduce HG-induced LDH leakage and MDA activity. Simultaneously, it was reported that VEGF plays an important role in promoting angiogenesis including tube formation [33]. Recent available studies indicated that HG-induced suppression of tube formation and proliferation were improved through IGF1R/AKT/VEGF signalling, as well as the expression of VEGF protein [34]. Moreover, the upregulation of VEGF enhanced cardiac angiogenesis following HG exposure in rats [35]. Studies demonstrated that Andr restrained cell migration in oral cancer cells [36], renal cell carcinoma 786-0 cells [37], and human glioblastoma multiforme cells [38]. Although some reports have indicated that Andr inhibits angiogenesis, our present research demonstrated that Andr promotes angiogenesis for the following possible reasons. First, a previous study [39] suggested that proliferation, cell migration, and tube formation are decreased in HUVECs in the Andr treatment group. In our study, Andr did not affect these phenotypes or affect the expression of VEGF. Second, our results show that Andr plays a protective role under HG conditions, so there may be several underlying mechanisms: (1) HG stimulation alters the chemotactic structure of Andr and promotes the release of VEGF and (2) HG stimulation alters the response of HUVECs to Andr, leading to phenotypic changes. Finally, a previous study investigated the damaging effects of Andr on cerebral endothelial cells. Although the dosage was similar to that in our study, different cells have different reactivities to Andr, which may lead to different phenotypes. Meanwhile, previous research demonstrated that with increasing Andr concentrations (5, 10, 20, 50, and 100 *μ*M), the damage to cerebral endothelial cells becomes more serious; however, 50 *μ*M Andr treatment exerted a protective effect on endothelial cells in our study. The reasons may be attributed to the use of different models (ischaemia/reperfusion vs. high glucose) and different cells (cerebral endothelial cells vs. HUVECs), in which Andr plays different roles. Here, we determined that Andr exerts a protective effect in HG-induced cell migration and impairment of angiogenesis, as Andr inhibited HUVEC migration, promoting tube formation, the upregulated expression of VEGF protein expression, and the downregulation of LDH and MDA levels. After being cocultured with LY294002, MK-2206, and L-NAME, Andr slightly decreased the release of LDH and MDA and cell migration ability and slightly restored tube formation ability and the levels of VEGF protein.

PI3K/AKT signalling is a central pathway that mediates various pathophysiological processes [16]. In diabetes, after the combination of insulin and growth factor receptor tyrosine kinases, PI3K is activated and generates phosphatidylinositol 3,4,5-trisphosphate, which induces the phosphorylation of AKT (p-AKT); p-AKT then activates endothelial nitric oxide synthase (eNOS) and produces NO. Hence, AKT and eNOS are considered the central factors in angiogenesis [18, 40]. The downregulation of TNF*α*, IL-6, and NF-*κ*B expression was ameliorated by insulin through PI3K/AKT signalling [17]. Inactivation of the PI3K/AKT pathway led to increased positive apoptotic cells in neonatal rat cardiomyocytes [18] and prostate cancer cells [41]. Additionally, targeting VEGF leads to the inactivation of the PI3K/AKT pathway to suppress angiogenesis [19]. Moreover, epithelial-mesenchymal transition triggers the activation of PI3K/AKT signalling, which is involved in the process of hepatocellular carcinoma cell migration [42]. According to these studies, we investigated the role of Andr in PI3K/AKT-eNOS signalling after exposure to HG. Our results demonstrated that HUVECs exposed to HG had remarkably reduced levels of p-PI3K, p-AKT, and p-eNOS protein; this effect was ameliorated by Andr treatment, which is consistent with the findings of a pervious study [20, 21]. Interestingly, the T-PI3K, T-AKT, and T-eNOS protein levels did not exhibit notable changes.

It has been reported that abnormal increases in ROS and reactive nitrogen species (RNS) induced by high glucose stimulation can damage proteins, carbohydrates, lipids, and nucleic acids, leading to oxidative/nitrosative stress. Excessive oxidation/nitrosative stress contributes to irreversible vascular injury, and HG-induced oxidative stress interferes with cell migration. This inhibition may be due to abnormal cell polarity, decreased adhesion maturity, and destabilization of protrusions [43]. In the present study, HG-induced cell migration was inhibited by Andr treatment. To the best of our knowledge, there are still significant differences in the reports of the specific mechanisms of Andr in cell migration. Andr suppresses HUVEC migration by inhibiting the expression of MMP-2 and MMP-9 [44]. Furthermore, Andr has the ability to inhibit MDA-MB-231 breast cancer cell migration by regulating nuclear factor-*κ*B-dependent matrix metalloproteinase-9 expression [45]. Based on these data, it is reasonable to conclude that inhibition of Andr plays a protective role in HG-induced cell migration.

Alteration of endothelial cell structure and function is the pathological basis for many cardiovascular diseases, such as hypertension and diabetic cardiomyopathy. HG is a common cause of endothelial dysfunction [46, 47]. In the present study, Andr could alleviate inflammation and apoptosis induced by HG, consequently improving vascular endothelial dysfunction. Thus, Andr may delay the progression of cardiovascular diseases, reduce their mortality, and improve the quality of life of patients.

**Figure 5:**
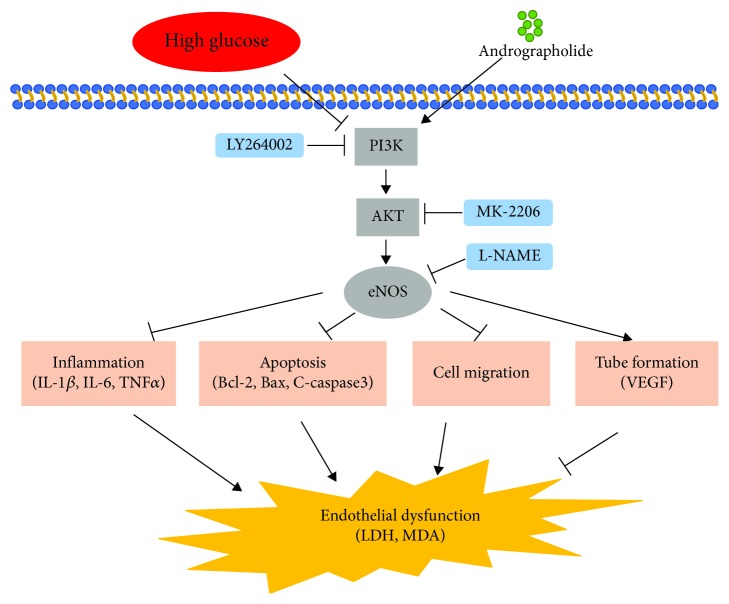
Graphical abstract. Andrographolide (Andr) attenuates HG-induced vascular endothelial dysfunction. Andr increases the expression of P-PI3K, P-AKT, and P-eNOS that was inhibited by HG, thus suppressing the gene expression of IL-1*β*, IL-6, and TNF*α* and the expression of VEGF. Furthermore, this effect inhibits HUVECs and promotes tubule formation, consequently decreasing the levels of LDH and MDA induced by HG.

Previous studies have shown that Andr inhibits inflammation by downregulating the activity of NF-*κ*B and NLRP3 expression [48]. Increasing the expression of STAT3 [49] and Nrf2 [50] can also make Andr play an anti-inflammatory role. Singha et al. [51] found that Andr reduces lipid peroxidation by activating the PI3K/AKT/AP-1 signalling pathway. The PI3K/AKT signalling pathway has been demonstrated to be involved in cell proliferation, migration, apoptosis, cell cycle, telomerase activity, and inflammation [16]. As shown in Figure 5, PI3K/AKT is an axis, and eNOS is a downstream of PI3K/AKT. Andr enters cells by acting on cytokine surface receptors. PI3K is activated by Andr, which further phosphorylates AKT and finally activates eNOS. Moreover, studies suggest that Andr can activate eNOS directly after it enters the cell membrane [52]. However, the specific mechanism of Andr in the PI3K/AKT-eNOS signalling pathway needs further experimental study.

## 5. Conclusion

*In vitro*, our research demonstrated that Andr attenuated HG-induced inflammation, apoptosis, migration, and impairment of angiogenesis, via a mechanism associated with the activation of the PI3K/AKT-eNOS pathway.

## Figures and Tables

**Table 1:** Primer sequences used for RT-PCR.

Genes (species)	Forward	Reverse
IL-1*β* (H)	GGCTGCTCTGGGATTCTCTT	ATTTCACTGGCGAGCTCAGG
IL-6 (H)	TTTTGGTGTTGTGCAAGGGTC	ATCGCTCCCTCTCCCTGTAA
TNF*α* (H)	TGGGATCATTGCCCTGTGAG	GGTGTCTGAAGGAGGGGGTA
GAPDH (H)	TTCACCACCATGGAGAAGGC	AGTGATGGCATGGACTGTGG

H: human.

## Data Availability

The data used to support the findings of this study are included within the article.
